# Covid-19 vaccine acceptance among individuals incarcerated in Connecticut state jails

**DOI:** 10.1186/s40352-023-00216-x

**Published:** 2023-03-13

**Authors:** Margaret L. Lind, Byron S. Kennedy, Murilo Dorion Nieto, Amy J. Houde, Peri Sosensky, Ryan Borg, Derek A. T. Cummings, Albert I. Ko, Robert P. Richeson

**Affiliations:** 1 Department of Epidemiology of Microbial Diseases, Yale School of Public Health, 60 College Street, New Haven, CT 06510 USA; 2 Connecticut Department of Correction, 24 Wolcott Hill Rd, Wethersfield, CT 06109 USA; 3 Department of Biology, University of Florida, 214 Bartram Hall, Gainesville, FL 32611 USA; 4 Emerging Pathogens Institute, University of Florida, 2055 Mowry Rd, Gainesville, FL 32610 USA; 5 Instituto Gonçalo Moniz, Fundação Oswaldo Cruz, Salvador, BA Brazil

**Keywords:** Vaccine, Jails, COVID-19, Correctional Facility Health

## Abstract

**Background:**

Vaccine hesitancy is common among incarcerated populations and, despite vaccination programs, vaccine acceptance within residents remains low, especially within jails. With the goal of assessing the Connecticut DOC’s COVID-19 vaccine program within jails we examined if residents of DOC operated jails were more likely to become vaccinated following incarceration than in the community. Specifically, we conducted a retrospective cohort analysis among people who spent at least one night in a DOC-operated jail between February 2 and November 8, 2021, and were eligible for vaccination at the time of incarceration (intake). We compared the vaccination rates before and after incarceration using an age-adjusted survival analysis with a time-varying exposure of incarceration and an outcome of vaccination.

**Results:**

During the study period, 3,716 people spent at least one night in jail and were eligible for vaccination at intake. Of these residents, 136 were vaccinated prior to incarceration, 2,265 had a recorded vaccine offer, and 479 were vaccinated while incarcerated. The age-adjusted hazard of vaccination following incarceration was significantly higher than prior to incarceration (12.5; 95% Confidence Intervals: 10.2–15.3).

**Conclusions:**

We found that residents were more likely to become vaccinated in jail than in the community. Though these findings highlight the utility of vaccination programs within jails, the low level of vaccination in this population speaks to the need for additional program development within jails and the community.

**Supplementary Information:**

The online version contains supplementary material available at 10.1186/s40352-023-00216-x.

## Background

COVID-19 has disproportionately affected people who experience incarceration in state or federal run correctional facilities (Hawks et al., 2020; Lemasters et al., 2020; Park, 2021; Sims et al., 2021; Wang et al., 2020). To mitigate transmission and disease burden, US state and federal Departments of Correction (DOCs) implemented vaccine programs in the winter of 2020–2021 (Hagan et al., 2021; Hawks et al., 2020; Liu et al., 2022). However, vaccine hesitancy is common among incarcerated populations due to distrust of the medical community and uncertainty around vaccine effectiveness (Liu et al., 2022; Ortiz-Paredes et al., 2022; Vicente-Alcalde et al., 2020). Consequently, data from state and federal correctional facilities suggest vaccine acceptance within residents is below that needed for population level control, especially within jails (Chin et al., 2021; Hagan et al., 2021; Khorasani et al., 2021; Liu et al., 2022; Stern, 2020). According to a study by Liu et al. vaccine acceptance within residents of California jails was less than 60% as of mid-summer 2021, far below that observed in the general population from the surrounding counties (84–86%) (Liu et al., 2022).

Despite the continued need for vaccine programs within jails, the impact vaccine programs have on overall vaccine acceptance within people who experience incarceration in jails is not well characterized. Herein we leveraged data from Connecticut-DOC-operated jails to evaluate the success of the DOC’s vaccination program within jails. Specifically, we calculated vaccine acceptance among residents with recorded vaccine offers. Further, to test if the program resulted in increased vaccination within this population, we compared vaccination rates among newly incarcerated people before and after incarceration.

## Methods

### Study sample and population

 The state of Connecticut began COVID-19 vaccine distribution in the community on January 14, 2021 (e.Table1). The Connecticut DOC initiated their COVID-19 vaccination program on February 2, 2021, directly following receipt of their state-supplied allotment, for residents of their 12 prisons (long-term post sentencing facilities) and four jails (pre or short-term post sentencing facilities). Under the program, residents who qualified for vaccination according to state-defined eligibility (e.Table 1) and were not currently infected with SARS-CoV-2 were offered vaccination by DOC medical staff at the time of incarceration, when visiting medical facilities, and during mass vaccine campaigns (periods when already housed residents were offered the vaccine). Additionally, residents who asked DOC staff for the vaccine were provided the vaccine if they met state-defined eligibility criteria. Residents who were partially vaccinated prior to incarceration were offered second doses of the corresponding vaccine. Residents were provided with the manufacturers’ information sheets at the time they were offered or asked for the vaccine. Additionally, CDC produced educational brochures and fliers were distributed throughout the facilities to encourage uptake.

Deidentified housing (facility, unit and bed), demographic, community vaccination history, and within-facility vaccine offer and acceptance data were extracted from DOC databases. The movement data was used to determine when residents were incarcerated and if they were incarcerated within a jail or prison. Community vaccinations were self-reported and the vaccination status of each resident was verified using the CT WiZ, Connecticut’s COVID-19 vaccine registry. Race/ethnicity data were self-reported. If racial classifications varied between datasets, the most frequently reported classification was selected. From these data, we identified people who spent at least one night in a DOC-operated jail while vaccine eligible between February 2 and November 8, 2021. We limited our analysis to residents whose first incarceration during the study was in jail and excluded data collected after a resident’s first discharge from jail.

### Measures and statistical analysis

#### Vaccine acceptance analysis

From these data, we estimated vaccine acceptance as the number of accepted doses over the number of recorded, offered doses. We stratified acceptance by first recorded offer and subsequent recorded offer and estimated acceptance by patient (age and race/ethnicity) and incarceration (date of incarceration relative to vaccine availability) factors.

#### Rate of vaccination before and after incarceration analysis

To compare vaccination rates before and after incarceration, we restricted our sample to residents who were vaccine eligible at the time of incarceration (intake), had not been vaccinated prior to February 2, 2021, and were incarcerated on or after February 2, 2021. We performed a survival analysis with a time varying exposure of incarceration and an outcome of vaccination (receipt of first dose). Follow-up time was defined as the interval between vaccine eligibility and the receipt of the first vaccine dose, death, or first discharge from jail, whichever occurred first. We visualized the difference in the probability of vaccination using Kaplan Meier curves and estimated age-adjusted hazard ratios (HR) using Cox Proportional Hazards models. Additionally, we generated race/ethnicity-specific (non-Hispanic Black, non-Hispanic White, Hispanic) Kaplan Meier curves and age-adjusted HRs. This work was determined to be a public health surveillance activity by the Yale University Institutional Review Board and exempt from review (ID: 200003233).

### Sensitivity analyses

We conducted four sensitivity analyses testing the robustness of our findings to alternative inclusion criteria. Specifically, we conducted analyses that included community vaccinations prior to February 2, restricted to residents vaccinated in the community or with recorded vaccine offers, restricted to residents who were vaccine eligible in the community for at least one week, and restricted to residents incarcerated on or after May 15 (Supplement).

## Results

Between February 2 and November 8, 2021, 6,522 people stayed at least one night in a DOC-operated jail while vaccine eligible and had not been previously incarcerated during the study period (median length of stay: 70 days; Interquartile Range [IQR]: 13–182 days).

### Vaccine Acceptance

**Fig. 1:**
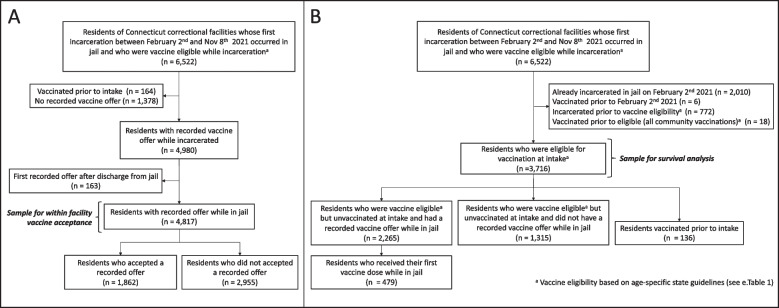
Flow Chart of Residents of Connecticut Department of Correction Operated Jails Between February 2, 2021 and November 8, 2022. Legend: Population flow chart of residents of Department of Correction (DOC) operated jails included in the **A** Vaccine Acceptance Examination and **B** Rate of Vaccination Before and After Incarceration Analysis. Vaccine eligibility was based on the residents birthdate and the date that their respective age group was eligible to receive the vaccine

Among the 6,358 residents who were unvaccinated at intake, 4,980 (78.3%) had a recorded vaccine offer (Fig. 1.A). Of the 4,817 residents whose first recorded offer occurred in jail, 24.9% (1,198/4,817) accepted after the first and 18.3% (664/3,619) accepted following subsequent recorded offers, resulting in an overall acceptance proportion of 38.7% (1,862/4,817). Vaccine acceptance was higher for non-Hispanic Whites and Hispanics than non-Hispanic Blacks and for residents incarcerated at the time of program rollout (February 2, 2021; Table 1).

**Table 1:** Recorded First Vaccine Offers and Acceptance Proportion for Residents Incarcerated in a Connecticut State Jail Between February 2, 2021, and November 8, 2021 by Demographic Characteristics

	**Total**	**Offered**	**Accepted First Offer**	**Accepted Subsequent Offer**
	**(*N* = 6522)**	**(*N* = 4817)**	**(*N* = 1198)**	**(*N* = 664)**
**Demographics**				
Age years, Median (IQR)	36 [18, 79]	35 [18, 79]	39 [18, 77]	36 [18, 74]
Race/Ethnicity, n (%)				
Hispanic/Latinx	1895 (29.1%)	1385 (28.8%)	401 (33.5%)	210 (31.6%)
Non-Hispanic Black	2596 (39.8%)	1964 (40.8%)	391 (32.6%)	263 (39.6%)
Non-Hispanic White	1979 (30.3%)	1432 (29.7%)	395 (33.0%)	186 (28.0%)
Other Race	52 (0.8%)	36 (0.7%)	11 (0.9%)	5 (0.8%)
Incarcerated prior to vaccine program implementation (Feb 2nd 2021)				
First incarceration event prior to Feb 2nd	2010 (30.8%)	1899 (39.4%)	747 (62.4%)	275 (41.4%)
First incarceration event after Feb 2nd prior to age-based eligibility	772 (11.8%)	653 (13.6%)	162 (13.5%)	111 (16.7%)
First incarceration event after Feb 2nd while eligibility	3740 (57.3%)	2265 (47.0%)	289 (24.1%)	278 (41.9%)

### Rate of vaccination before and after incarceration

Of the 3,716 residents who were eligible for vaccination at intake (Fig. 1.B), 136 (3.7%) were vaccinated prior to incarceration, 2,265 (61.0%) had a recorded offer and 479 (12.9%) became vaccinated while in jail. Residents spent more time eligible for vaccination in the community (79 days [IQR: 41–183]) than in jail (14 days [IQR: 3–31]; Table 2).

**Table 2:** Demographic and Vaccination Characteristics of Residents Incarcerated in Jail on the Day of or Following Statewide, Age-Specific Vaccine Eligibility

	**Total**	**Unvaccinated**^a^	**Vaccinated in Community**	**Vaccinated in Facility**
	**(*N* = 3716)**	**(*N* = 3101)**	**(*N* = 136)**	**(*N* = 479)**
**Demographics**				
Age years, Median (IQR)	35 [28, 45]	34 [27, 44]	41 [33, 52]	38 [32, 48]
Race/Ethnicity, n (%)				
Hispanic/Latinx	1069 (28.8%)	888 (28.6%)	40 (29.4%)	141 (29.4%)
Non-Hispanic Black	1395 (37.5%)	1173 (37.8%)	46 (33.8%)	176 (36.7%)
Non-Hispanic White	1217 (32.8%)	1012 (32.6%)	49 (36.0%)	156 (32.6%)
Other Race	35 (0.9%)	28 (0.9%)	1 (0.7%)	6 (1.3%)
**Eligible Time**				
Time in community, median (IQR)	79 [41, 183]	90 [44, 185]	50 [33, 89]	58 [34, 104]
Time in jail, median (IQR)	14 [3, 31]	14 [4, 34]	0 [0, 0]	10 [5, 31]

^a^Remained unvaccinated as of November 8th, 2021, or upon departure from DOC facility

**Fig. 2:**
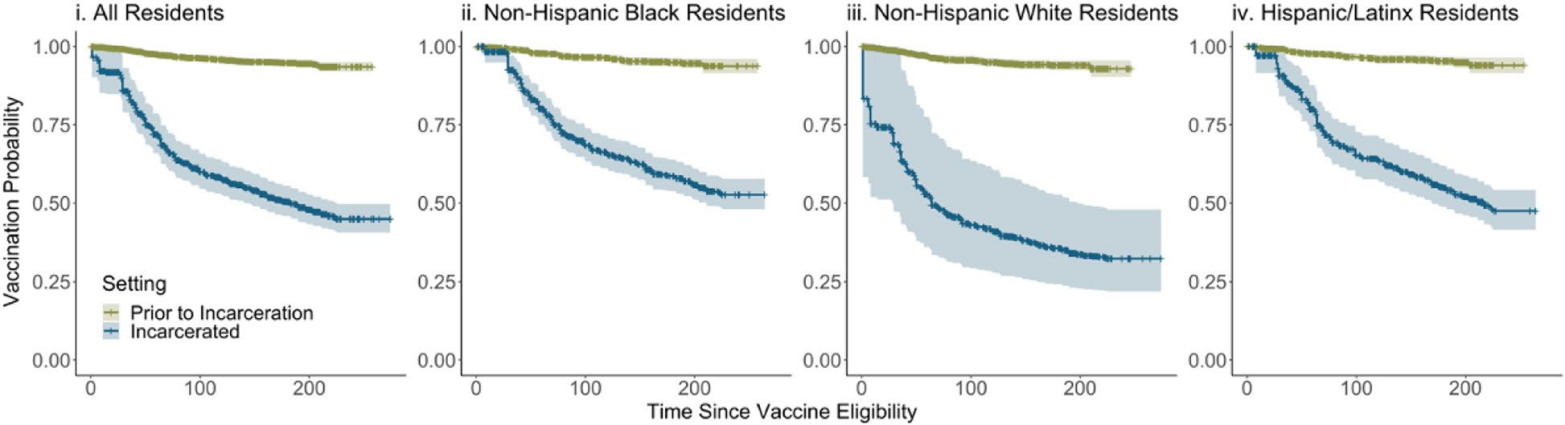
Vaccination Within Newly Incarcerated Residents of Connecticut State Operated Jails Before and After Incarceration. Legend: Kaplan Meier curves displaying the probability of vaccination among newly incarcerated residents of Connecticut State jails who were eligible for vaccination on the day of their incarceration. Plots are faceted by included resident population: (i) All residents, (ii) Non-Hispanic Black residents, (iii) Non-Hispanic White residents, (iv) Hispanic/Latinx residents

**Fig. 3:**
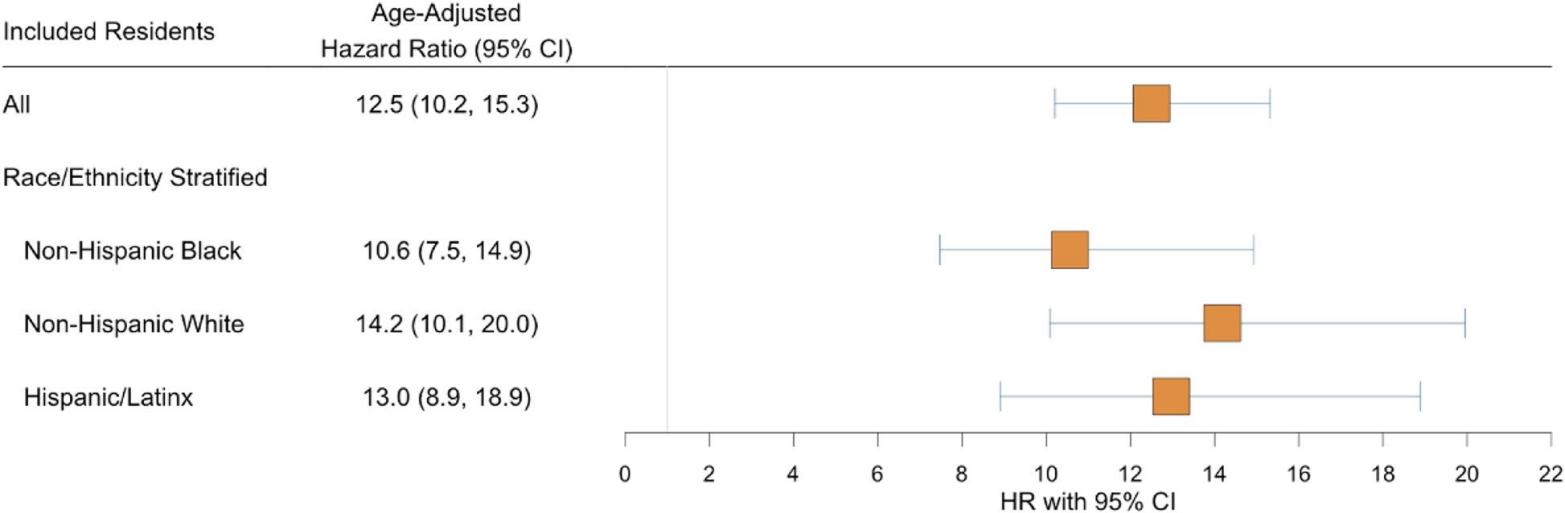
Forest Plot Comparing the Age-Adjusted Hazard Ratios of Vaccination Before and After Incarceration. Legend: Forest plot of age-adjusted hazard ratios compare instantaneous vaccination before and after incarceration. Vaccine eligibility was based on the individual’s birthdate and the date that their respective age group was eligible to receive the vaccine

The probability of vaccination was higher following incarceration than prior to incarceration across all residents and among non-Hispanic Black, non-Hispanic White, and Hispanic residents (Fig. 2). Following adjustment for age, the hazard of vaccination was 12.5 (95% Confidence Intervals [CI]: 10.2–15.3) times higher following incarceration than prior to incarceration. The HR was highest for non-Hispanic Whites (14.2 [95% CI: 10.1–20.0]) and lowest for non-Hispanic Blacks (10.6 [95% CI: 7.5–14.9]; Fig. 3). Global Schoenfeld Residual *p*values were > 0.05 (e.Figs. 1–4) (Hess, 1995).

### Sensitivity analyses

The age-adjusted HRs were significant for each sensitivity analysis (e.Tables 2–5). The HR was highest when we restricted to residents incarcerated on or after May 15 (22.2, e.Table 2) and lowest when we restricted to residents vaccinated in the community or with recorded vaccine offers (7.0, e.Table 3). For each examined scenario, the race stratified HR for non-Hispanic Blacks had the lowest HR (range: 5.5–18.6; e.Tables 2–5).

## Discussion

Vaccine programs were implemented within correctional facilities to help mitigate the risk of COVID-19 among residents (Hagan et al., 2021; Hawks et al., 2020; Liu et al., 2022). Despite the implementation of such programs, vaccine acceptance within facilities remains low relative to general US residents, especially within jails (CDC, 2020; Liu et al., 2022; Stern, 2020). In our evaluation of the Connecticut DOC’s vaccine program within jails, we observed a slightly lower vaccine acceptance proportion than has been reported for residents of jails from other US regions (ours: 38.7%, previously reported: 40.9–56.7%) (Chin et al., 2021; Khorasani et al., 2021; Liu et al., 2022; Stern, 2020). Further the observed acceptance proportion was meaningfully lower than general Connecticut residents (Connecticut residents 16 years of age or older who received at least one vaccine dose as of June 2, 2021: 71%; as of November 10, 2021: 86%) (Connecticut Department of Public Health 2022).

The strength of the Connecticut DOC’s vaccination program is evidenced by the results of our survival analysis among people who become incarcerated following vaccine eligibility. Among this population, we found that following the same amount of time spent eligible for the vaccine, people currently incarcerated were 12.5 times more likely to initiate vaccination than people prior to incarceration. Though our findings highlight the ability of within-facility vaccine programs to improve vaccine coverage among this population, the moderate and racially imbalanced vaccine acceptance proportion points to the need for ongoing, evidence-based vaccination program development.

While not examined here, prior research examining vaccine hesitancy within marginalized populations suggests that vaccine acceptance among residents of correctional facilities may be improved by building trust between residents and healthcare providers, reducing or eliminating financial barriers, and providing incentives (Liu et al., 2022; Murphy et al., 2021; Ortiz-Paredes et al., 2022; Stern, 2020; Strully et al., 2021). Additionally, acceptance within residents may be increased through educational campaigns focused on vaccine effectiveness and side effects (Liu et al., 2022; Murphy et al., 2021; Ortiz-Paredes et al., 2022; Stern, 2020; Strully et al., 2021). Educational campaigns should leverage the community infrastructure by employing family members or influential community members (Giuseppe et al., 2022; Liu et al., 2022; Ortiz-Paredes et al., 2022; Stern, 2020).

Our results also speak to the need for evidence-based community program development outside of jails. This stems from the low level of vaccination among our included residents prior to incarceration. As with within facility program development, programs should employ accessible and trusted sources of information, such as friends and family, to address vaccine-related concerns and foster trust in medical personnel (Giuseppe et al., 2022; Liu et al., 2022; Stern, 2020).

### Limitations

Our analysis was subject to limitations. First, not all within facility vaccine offers are recorded, and our acceptance numbers are based solely on recorded offers. Second, our vaccination rates are based on recorded doses from the facilities and CT WiZ, both of which are subject to potential data entry errors and missingness. Third, the DOC racial data were subject to reporting inconsistencies (self-reported). Finally, our selection of residents and at-risk time for the survival analysis may have introduced bias by incorporating unequitable amounts of person time in the community and following incarceration. While the sensitivity analyses highlight the robustness of our findings to such bias, the magnitude of the observed association varied by examined sample.

## Conclusions

Despite observing moderate levels of vaccine acceptance among residents of state jails, we found newly incarcerated persons were far more likely to initiate vaccination following incarceration than prior to incarceration. Though our findings highlight the utility of vaccination programs within correctional facilities, they also speak to the need for ongoing, evidence-based vaccination program development within correctional facilities and the community.

## Supplementary Information

**Additional file 1.** Supplemental Appendix.

## Data Availability

The data used in this study belongs to the Connecticut Department of Correction. Qualified researchers may submit a data share request for de-identified patient level data by contacting the corresponding author with a detailed description of the research question.
